# Radiation-Based Multimodal Strategies for Esophageal Squamous Cell Carcinoma: From Definitive Chemoradiotherapy to Salvage Treatment

**DOI:** 10.3390/cancers18111681

**Published:** 2026-05-22

**Authors:** Yusuke Taniyama, Keiichi Jingu, Chiaki Sato, Hiroshi Okamoto, Yohei Ozawa, Hirotaka Ishida, Naoto Ujiie, Michiaki Unno, Takashi Kamei

**Affiliations:** 1 Department of Surgery, Graduate School of Medicine, Tohoku University, 2-1 Seiryo-machi, Aoba-ku, Sendai 980-8575, Miyagi, Japan; 2 Department of Radiation Oncology, Graduate School of Medicine, Tohoku University, 1-1 Seiryo-chou, Aoba-ku, Sendai 980-8574, Miyagi, Japan

**Keywords:** esophageal squamous cell carcinoma, definitive chemoradiotherapy, salvage surgery

## Abstract

Esophageal squamous cell carcinoma (ESCC) is a highly aggressive malignancy in which radiotherapy plays a uniquely central role compared with other gastrointestinal cancers. Definitive chemoradiotherapy (dCRT) is widely used as a curative treatment; however, a substantial proportion of patients develop residual or recurrent disease, creating a complex clinical scenario that requires tailored salvage strategies. Salvage esophagectomy offers the potential for long-term survival but remains technically demanding and is associated with significant morbidity because of radiation-induced tissue damage. Less invasive local therapies, such as endoscopic submucosal dissection and photodynamic therapy, may provide effective treatment in selected patients, although their indications are limited by tumor characteristics and post-radiation fibrosis. In addition, immune checkpoint inhibitors have demonstrated promising efficacy in advanced ESCC and may represent a potential therapeutic option in the salvage setting. For patients who are not candidates for curative treatment, palliative esophageal stenting remains an important option for symptom relief, although prior radiotherapy may increase the risk of treatment-related complications. Given the diversity of available treatment modalities and their associated risks, a multidisciplinary and individualized treatment approach is essential. Further prospective studies are warranted to optimize treatment algorithms and improve outcomes in patients with ESCC after dCRT.

## Simple Summary

Esophageal squamous cell carcinoma is an aggressive cancer for which radiotherapy plays a central role in treatment. Definitive chemoradiotherapy is widely used as a curative option; however, many patients develop residual or recurrent disease after treatment. Managing these cases remains clinically challenging. This review summarizes current treatment strategies for such patients, including salvage surgery, endoscopic treatments, photodynamic therapy, immunotherapy, and palliative stenting. Each approach has specific benefits and risks, particularly in the context of prior radiation therapy, which can complicate subsequent treatment. Recent advances in minimally invasive techniques and immunotherapy have expanded therapeutic options. A multidisciplinary and individualized approach is essential to achieve the best possible outcomes. This review provides an overview of current evidence and highlights future directions in the management after definitive chemoradiotherapy.

## 1. Introduction

Surgical resection has long been considered the cornerstone of curative cancer treatment. Across a wide spectrum of solid malignancies, surgery remains the most reliable modality for achieving complete tumor eradication and long-term survival. Esophageal cancer is no exception; esophagectomy continues to play a central role in curative-intent therapy and remains the standard treatment for resectable disease [1].

However, esophagectomy is among the most invasive procedures in gastrointestinal surgery, requiring extensive dissection within the thoracic and abdominal cavities, often combined with complex reconstruction. Despite advances in perioperative management, anesthesia, and minimally invasive surgical techniques, esophagectomy is still associated with substantial morbidity and a non-negligible risk of mortality compared with other gastrointestinal malignancies. Postoperative complication rates exceed 50%, and mortality rates are generally reported to range from approximately 2% to 5%, even in high-volume centers [2,3]. These concerns are particularly relevant in patients with limited physiological reserve, who may not tolerate such an aggressive surgical approach.

In contrast to many other gastrointestinal cancers, esophageal squamous cell carcinoma (ESCC), which frequently arises in the thoracic esophagus, has historically been treated with radiotherapy as a definitive modality [4,5]. Definitive chemoradiotherapy (dCRT) has therefore become established not only as a neoadjuvant treatment [6] but also as an alternative curative strategy, particularly for patients who are medically inoperable or who decline surgery. Even in operable cases, dCRT may represent a viable treatment option depending on patient preference and tumor characteristics. In ESCC, radiotherapy is not merely a complementary treatment modality but a central platform upon which multimodal treatment strategies are constructed.

More recently, the advent of immune checkpoint inhibitors (ICIs) has dramatically reshaped the treatment landscape of esophageal cancer. Landmark clinical trials, including the CheckMate and KEYNOTE series [7,8], have demonstrated significant survival benefits of ICIs in patients with unresectable or advanced esophageal cancer. These agents have shown durable antitumor activity and expanded systemic treatment options beyond conventional cytotoxic chemotherapy. Furthermore, accumulating evidence has established the efficacy of ICIs in the adjuvant setting [9].

Despite these advances, radiotherapy continues to play a uniquely important role in ESCC, distinguishing it from other gastrointestinal malignancies in which radiotherapy is less commonly used. Importantly, definitive radiotherapy creates a distinct clinical scenario involving the management of residual or recurrent disease after radiation-based treatment. In this review, we focus on salvage strategies after dCRT for ESCC, including surgical, endoscopic, immunotherapeutic, and palliative approaches, and discuss their roles within contemporary multimodal treatment strategies. By reviewing current evidence and emerging therapeutic concepts, we aim to provide insight into future directions for optimizing the management of this complex disease.

## 2. Definitive Chemoradiotherapy in ESCC

For locally or regionally advanced disease, neoadjuvant chemotherapy with docetaxel, cisplatin, and 5-fluorouracil (DCF) followed by surgery is considered the standard treatment strategy in Japan. In patients who are not suitable candidates for surgery, dCRT is often selected as an alternative curative approach.

The foundation of modern dCRT was established by the landmark Radiation Therapy Oncology Group (RTOG) 85-01 trial, which compared radiotherapy alone with concurrent chemoradiotherapy consisting of cisplatin and 5-fluorouracil. This randomized trial demonstrated a significant survival benefit with combined treatment, with a 5-year overall survival rate of approximately 26%, whereas no 5-year survivors were observed in the radiotherapy-alone group [4]. Following RTOG 85-01, several studies investigated strategies to improve treatment outcomes, including radiation dose escalation and induction chemotherapy. The INT 0123 (RTOG 94-05) trial compared standard-dose radiotherapy (50.4 Gy) with high-dose radiotherapy (64.8 Gy) administered concurrently with chemotherapy [5]. However, higher radiation doses did not improve survival outcomes and were associated with increased treatment-related toxicity. Consequently, concurrent chemotherapy with approximately 50–50.4 Gy remains the widely accepted standard dCRT regimen.

A randomized comparative study conducted in China comparing 50.4 Gy with 59.4 Gy using intensity-modulated radiotherapy (IMRT) also demonstrated no significant differences, and 50.4 Gy has become established as the standard radiation dose in China [10]. In addition, a meta-analysis of four randomized controlled trials published in 2023 revealed that higher radiation doses of 60–66 Gy were associated with increased adverse events without improvements in local control or overall survival compared with standard-dose radiotherapy of 50–50.4 Gy [11]. Despite these advances, locoregional recurrence remains a significant clinical problem, with reported failure rates of approximately 40–60% after dCRT. These findings highlight the need for improved local salvage strategies.

**Figure 1:**
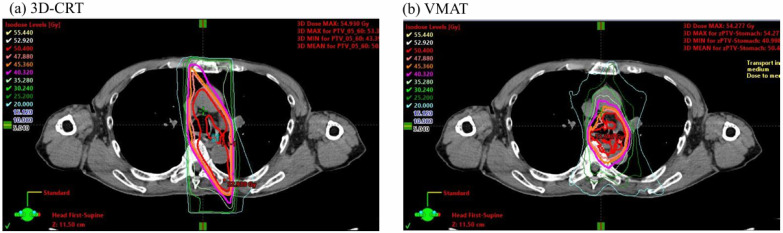
Comparison of isodose distribution between three-dimensional conformal radiotherapy (3D-CRT) and volumetric modulated arc therapy (VMAT) in the same patient. Both plans delivered 50.4 Gy in 28 fractions. (**a**) Conventional 3D-CRT using a four-field arrangement, showing a broader high-dose distribution to the planning target volume (PTV). (**b**) VMAT demonstrating improved target conformity, although with broader low-dose irradiation to surrounding tissues.

Even with three-dimensional conformal radiotherapy (3D-CRT), multi-field irradiation techniques have increasingly been adopted for relatively localized clinical target volumes (CTVs) to reduce late cardiac toxicity. More recently, as described above, prophylactic nodal irradiation has often been omitted, and IMRT, including volumetric modulated arc therapy (VMAT), has increasingly been introduced into clinical practice (Figure 1). Meta-analyses and large retrospective studies from Asia have suggested that IMRT may provide improved survival outcomes compared with 3D-CRT [12,13,14].

In dCRT, concurrent chemotherapy with cisplatin and 5-fluorouracil is considered the standard regimen. In recent years, favorable outcomes have also been reported with the combination of DCF and radiotherapy; however, caution is warranted because of the high incidence of treatment-related adverse events [15]. In patients who are intolerant to cisplatin, oxaliplatin, leucovorin, and 5-fluorouracil (FOLFOX) may serve as an alternative regimen [16]. Beyond the definitive setting, ICIs have become an essential component of first-line systemic therapy for advanced esophageal cancer, with recent meta-analyses demonstrating significant survival benefits, particularly in PD-L1-positive ESCC [17]. Furthermore, combining immunotherapy with dCRT is expected to improve treatment outcomes, although clinical investigation in patients with unresectable locally advanced esophageal cancer remains at an exploratory stage.

The KEYNOTE-975 trial, a pivotal randomized phase III study, is currently evaluating pembrolizumab in combination with dCRT in patients with unresectable locally advanced esophageal cancer [18]. Approximately 600 patients with either squamous cell carcinoma or adenocarcinoma are being randomized to receive pembrolizumab or placebo concurrently with dCRT, followed by maintenance therapy. The results of KEYNOTE-975 are expected to clarify whether the addition of PD-1 blockade to dCRT can improve outcomes in locally advanced esophageal cancer. In addition, the EC-CRT-002 phase II trial demonstrated that tislelizumab combined with induction chemotherapy and concurrent chemoradiotherapy achieved promising survival outcomes with acceptable toxicity in unresectable locally advanced ESCC, highlighting the potential role of immunotherapy-based treatment intensification in this setting [19]. Exploratory analyses also suggested a possible association between PD-L1 expression and treatment efficacy, although further validation is required.

## 3. Advances in Radiation Therapy Techniques

Radiation therapy techniques for ESCC have evolved substantially over the past two decades, resulting in improved dose conformity and reduced toxicity to surrounding normal tissues.

Historically, 3D-CRT was the standard radiation delivery technique. However, because of the anatomical location of the esophagus within the thoracic cavity, adjacent organs such as the lungs, heart, and spinal cord are often exposed to significant radiation doses. Consequently, treatment-related toxicities, including radiation pneumonitis and cardiac complications, remain important clinical concerns.

The introduction of IMRT has markedly improved radiation delivery for esophageal cancer. IMRT enables highly conformal dose distributions by modulating beam intensity across multiple radiation fields, thereby improving target coverage while sparing surrounding organs at risk. Several retrospective and population-based studies have suggested that IMRT may reduce cardiopulmonary toxicity and improve survival outcomes compared with 3D-CRT in patients undergoing dCRT [12,13,14,20,21]. However, it should be noted that although the current evidence includes a systematic review and meta-analysis, as well as several retrospective propensity score-matched and population-based studies, no randomized controlled trials have directly compared IMRT with 3D-CRT in this setting.

Another promising development is proton beam therapy (PBT). Proton therapy offers unique dosimetric advantages because of the Bragg peak phenomenon, which allows most of the radiation dose to be deposited within the tumor while minimizing the exit dose. Dosimetric studies have demonstrated that PBT can significantly reduce radiation exposure to the heart and lungs compared with photon-based radiotherapy [22]. More recently, a phase IIb randomized trial from MD Anderson Cancer Center comparing IMRT with PBT demonstrated no significant differences in progression-free or overall survival; however, the posterior mean total toxicity burden (TTB) was 2.3-fold higher with IMRT (39.9%) than with PBT (17.4%). Furthermore, the mean postoperative complication score was 7.6-fold higher in the IMRT group (19.1 vs. 2.5) [23].

Image-guided radiotherapy (IGRT) has further improved treatment accuracy by enabling daily verification of tumor positioning. Techniques such as cone-beam CT allow clinicians to account for interfractional anatomical changes and ensure precise dose delivery. In addition, four-dimensional CT (4D-CT) imaging enables assessment of respiratory motion, thereby facilitating treatment planning that accounts for tumor movement during respiration. These advances have allowed reduction in planning target volume margins and minimization of radiation exposure to healthy tissues.

Emerging technologies such as online adaptive radiotherapy (oART) are also attracting increasing attention. Adaptive radiotherapy involves modification of treatment plans during the course of therapy in response to anatomical changes, including tumor shrinkage and patient weight loss. By adapting radiation plans according to daily imaging findings, oART may further improve target coverage while reducing radiation exposure to surrounding normal tissues. Although clinical evidence remains limited, this approach has the potential to improve treatment precision and enhance the therapeutic ratio. Although advanced radiotherapy techniques such as 4D-CT and PBT may improve treatment precision and reduce radiation-related toxicity, their broader application is often constrained by cost and limited accessibility. In particular, PBT remains available only in specialized centers because of its high infrastructure and operational costs. Further studies are needed to clarify their cost-effectiveness and optimize their clinical utilization.

## 4. Standard Treatment Outcomes by dCRT

A multicenter clinical study evaluating patients treated with dCRT or radiotherapy alone between 2004 and 2008 reported 5-year survival rates of 73% for stage I disease, 40% for resectable stage II–III disease, and 18% for unresectable T4 or M1 lymph node-positive disease [24]. The JCOG9708 phase II trial investigated chemoradiotherapy for resectable stage I ESCC and demonstrated a 4-year overall survival rate of 80.5% [25].

Based on these favorable results, the JCOG0502 trial was conducted to compare outcomes between surgery and chemoradiotherapy. Although the randomized portion of the study was terminated early because of insufficient patient enrollment, the non-randomized cohort demonstrated the non-inferiority of dCRT. In the dCRT group, the 5-year overall survival rate was 85.5%, while esophageal preservation rates were 88.7% at 3 years and 80.4% at 5 years, indicating excellent organ preservation outcomes [26].

The JCOG0909 phase II trial evaluated dCRT with or without salvage surgery in patients with resectable stage II/III ESCC and reported a 3-year overall survival rate of 74.2% and a 3-year esophageal preservation survival rate of 63.6% [27]. In addition, the concept of conversion surgery, in which initially unresectable T3br or T4 tumors undergo surgical resection after becoming resectable during treatment, has attracted increasing attention. The ongoing JCOG1510 trial is currently evaluating this strategy, and previous studies have suggested that achievement of R0 resection may contribute to long-term survival [28].

The major landmark clinical trials related to dCRT and multimodal treatment strategies for ESCC are summarized in Table 1.

## 5. Patterns of Recurrence After Definitive Chemoradiotherapy

Despite advances in combined modality therapy, recurrence remains a major challenge after dCRT for ESCC. Both locoregional and distant failures are common, although their relative frequencies vary according to patient selection, treatment response, and disease stage. In a retrospective analysis of 302 patients treated with dCRT, including 204 patients who achieved complete response, Sudo et al. reported luminal relapse in 14%, regional lymph node recurrence in 6%, and distant metastasis in 19% of patients. In addition, second primary esophageal cancers and other malignancies developed in 17% and 8% of patients, respectively, during follow-up. Importantly, 93% of luminal relapses occurred within 3 years, and all regional recurrences were detected within 2 years after treatment [29].

In broader cohorts including all patients undergoing dCRT regardless of treatment response, distant metastasis represents the most common initial failure pattern. In a study of 276 patients treated with dCRT at MD Anderson Cancer Center, first relapse occurred as isolated local recurrence in 23.2% of patients, distant metastasis with or without local recurrence in 43.5%, and no recurrence in 33.3% during follow-up [30]. At final analysis, isolated local recurrence was observed in 14.5% of patients, distant metastasis alone in 15.9%, and combined local and distant relapse in 36.2%, indicating that systemic progression frequently develops during the disease course. In patients with more advanced or unresectable disease, locoregional recurrence may remain the dominant failure pattern. Therefore, achieving durable local tumor control continues to represent a critical therapeutic challenge in advanced ESCC.

## 6. Radiation-Associated Toxicities and Management

### 6.1. Adverse Events in the Acute Phase

**Figure 2:**
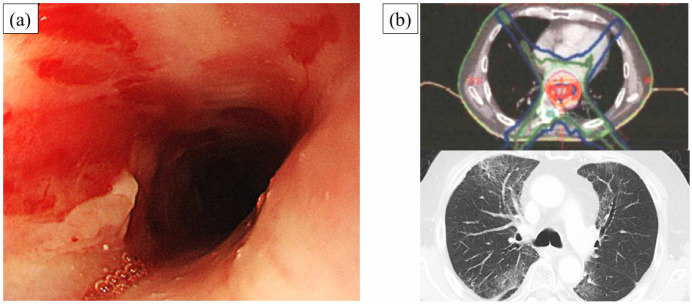
Radiation-Associated Toxicities. (**a**) radiation esophagitis, 1 months after radiation, (**b**) radiation pneumonitis, corresponding to the VMAT irradiation field. (VMAT: Volumetric Modulated Arc Therapy).

Radiation dermatitis, radiation esophagitis, hematologic toxicity, impaired renal function and radiation pneumonitis are representative side effects. In most cases, these adverse events are manageable and improve after completion of treatment. The most common acute toxicity during dCRT is radiation-induced esophagitis, which results from inflammation and mucosal injury of the esophagus (Figure 2a). Patients frequently experience odynophagia, dysphagia, and reduced oral intake, which may lead to significant weight loss and treatment interruption. The INT 0123 (RTOG 94-05) trial reported grade ≥ 3 esophagitis in 18–22% of patients, and Japanese studies have demonstrated comparable rates. In JCOG0502, grade ≥ 3 esophagitis occurred in approximately 10% of patients treated with concurrent cisplatin, 5-fluorouracil, and radiotherapy. Management generally consists of supportive care, including analgesics, proton pump inhibitors, and nutritional support. Hematologic toxicity is also common because of concurrent chemotherapy. In JCOG0502, grade ≥ 3 leukopenia, neutropenia, anemia, and thrombocytopenia occurred in 17%, 12%, 6%, and 3% of patients, respectively.

Radiation pneumonitis represents one of the most clinically important subacute toxicities associated with thoracic radiotherapy (Figure 2b). The reported incidence of symptomatic radiation pneumonitis (grade ≥ 2) after chemoradiptherapy for esophageal cancer ranges from 10% to 20%, whereas severe pneumonitis (grade ≥ 3) occurs in approximately 5–10% of patients. Several studies have suggested that IMRT may reduce the incidence of severe radiation pneumonitis, with reported grade ≥ 3 pneumonitis rates of approximately 5% [19].

Tracheoesophageal fistula is a rare but potentially fatal complication, with a reported incidence of approximately 2–5% following dCRT. This complication occurs more frequently in patients with locally advanced tumors invading the airway.

### 6.2. Adverse Events in the Late Phase

Late toxicities are less common than acute adverse events but remain clinically important. Reported risk factors include higher radiation dose, circumferential tumor involvement, and severe acute esophagitis during treatment. Clinicians should be aware of late complications such as pulmonary fibrosis, pleuritis, pericarditis, cardiovascular disease, myocardial injury, hypothyroidism, and esophageal stenosis. Esophageal stenosis requiring intervention has been reported in approximately 7–8% of patients and is considered to reflect late radiation-induced fibrosis [31]. In addition, previous studies have suggested that combining stent placement with definitive radiotherapy is associated with a high incidence of severe complications and poorer survival outcomes; therefore, stent insertion before definitive radiotherapy should generally be avoided [32]. However, in patients with limited life expectancy, a meta-analysis demonstrated that the addition of palliative radiotherapy after stent placement may prolong median survival [33].

Cardiac toxicity has increasingly been recognized as an important late complication of thoracic radiotherapy. Long-term follow-up studies have reported clinically significant cardiac events in approximately 10–15% of patients treated with chemoradiotherapy for esophageal cancer. Dose–volume parameters, including mean heart dose and heart V30, have been associated with an increased risk of cardiac morbidity. Studies comparing IMRT with 3D-CRT have demonstrated that IMRT significantly reduces cardiac radiation exposure and may decrease the incidence of cardiac complications. Similarly, PBT has shown favorable dosimetric advantages in reducing radiation dose to the heart and lungs, which may potentially translate into lower toxicity rates, although long-term prospective data remain limited.

In addition, the use of immunotherapy in esophageal cancer is associated with immune-related adverse events (irAEs). However, current evidence has not demonstrated an increased incidence of severe adverse events in patients with a history of thoracic radiotherapy [34]. Nomura et al. also suggested that the combination of immunotherapy and radiotherapy may be feasible and tolerable in this setting [35,36].

## 7. Salvage Surgery After Definitive Chemoradiotherapy

### 7.1. Indications for Salvage Surgery

A substantial proportion of patients experience either persistent disease or locoregional recurrence after dCRT, with reported rates ranging from approximately 40% to 60% [4,5]. However, treatment failure after dCRT should not be regarded as the end of therapeutic options, but rather as a transition point toward individualized salvage strategies. In such situations, salvage esophagectomy represents a potentially curative treatment option and should be considered in appropriately selected patients.

Patients with residual tumor after completion of dCRT, as well as those who develop isolated locoregional recurrence without distant metastasis, are generally considered candidates for salvage surgery [1]. Careful restaging using endoscopy, contrast-enhanced computed tomography, and positron emission tomography is essential to confirm the absence of systemic disease and assess resectability. However, evaluating resectability after radiotherapy remains challenging. Radiation-induced fibrosis, edema, and inflammatory changes frequently obscure normal anatomical planes, making accurate assessment of tumor invasion into adjacent vital structures such as the trachea, bronchus, and aorta difficult using conventional imaging techniques [37,38].

In particular, distinguishing true tumor invasion from post-treatment fibrotic adhesion is often unreliable on computed tomography, potentially leading to both overestimation and underestimation of T4 disease. Several alternative diagnostic modalities, including magnetic resonance imaging (MRI) and endoscopic ultrasonography (EUS), have been investigated to improve diagnostic accuracy in this setting. EUS may provide additional information regarding tumor depth and residual wall-layer structure [39], whereas MRI may better characterize soft tissue contrast and treatment-related changes [40]. However, current evidence remains limited, and no standardized or universally accepted diagnostic criteria have been established for evaluating local invasion after dCRT.

Patient selection remains critically important, as dCRT itself can impair physiological reserve. Radiation-induced toxicities, including radiation pneumonitis and pericardial effusion, may significantly compromise cardiopulmonary function and increase perioperative risk [41]. In addition, malnutrition and sarcopenia are frequently observed after dCRT and have been associated with poorer postoperative outcomes [42]. Therefore, the decision to perform salvage esophagectomy should be individualized and ideally determined within a multidisciplinary framework.

### 7.2. Surgical Considerations and Technical Challenges

**Figure 3:**
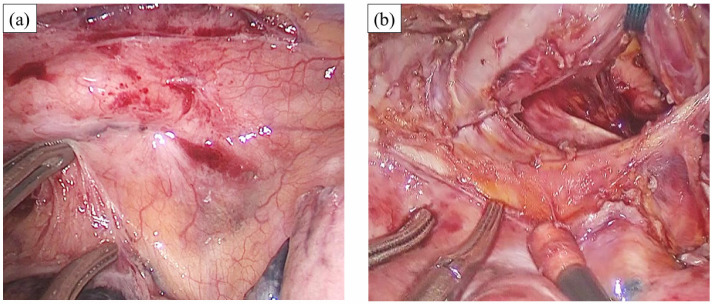
Salvage esophagectomy. (**a**) edematous change around esophagus, dissection between right bronchus and subcarinal lymph node, (**b**) strong adhesion by fibrotic change between trachea and esophagus.

Salvage esophagectomy is widely recognized as one of the most technically demanding procedures in esophageal surgery. Prior radiotherapy and chemotherapy induce substantial changes within the operative field, including dense fibrosis, tissue edema, and obliteration of normal anatomical planes [37,38] (Figure 3). These changes are particularly pronounced in the mediastinum, where dissection around vital structures such as the trachea, bronchi, and aorta becomes extremely challenging. Radiation-induced fibrosis results in firm adhesions between the esophagus and adjacent organs, frequently necessitating meticulous sharp dissection. In addition, tissue fragility and reduced elasticity further increase the risk of intraoperative injury, while vascular structures may be more susceptible to damage because of radiation-associated endothelial injury. Given these considerable technical challenges and operative risks, salvage esophagectomy should be performed by experienced surgeons, preferably at high-volume centers with specialized expertise in esophageal cancer management.

### 7.3. Complications

Salvage esophagectomy is associated with significantly higher morbidity and mortality than primary esophagectomy. Reported postoperative complication rates exceed 50%, while mortality rates range from 5% to 15%, even in high-volume centers [37,38,43,44,45,46].

Postoperative complications are frequently related to radiation-induced microvascular damage. Impaired tissue perfusion contributes to a higher incidence of anastomotic leakage, with reported rates of 20–40% [37,38,44,45,46]. Pulmonary complications, including pneumonia and respiratory failure, are also common and represent major causes of postoperative morbidity and mortality [44,45]. Among the most severe complications is tracheal or bronchial necrosis, a rare but often fatal condition. This complication is thought to result from compromised bronchial arterial blood supply in previously irradiated tissues [38]. These postoperative complications have been associated with significantly worse long-term outcomes and prognosis [45,47].

### 7.4. Strategies to Reduce Risk

Given the high-risk nature of salvage esophagectomy, meticulous perioperative management is essential.

Preoperative optimization plays a crucial role. Nutritional assessment and intervention are strongly recommended, as malnutrition is associated with increased postoperative complications [42]. Prehabilitation programs, including respiratory training and physical conditioning, may improve functional status and surgical tolerance, thereby helping to reduce postoperative respiratory complications [48]. Smoking cessation is also strongly recommended, ideally at least 2 months before surgery, to improve not only short-term surgical outcomes but also long-term prognosis after esophagectomy [49].

From a surgical perspective, minimizing operative invasiveness while maintaining oncological radicality is important. Several studies have suggested that minimally invasive esophagectomy (MIE) may reduce postoperative complications without compromising oncological outcomes [50,51]. Although radical lymph node dissection is considered standard during primary esophagectomy, accumulating evidence suggests that a more limited and selective lymphadenectomy may be appropriate in salvage settings to reduce operative morbidity and potentially improve prognosis [52,53]. In addition, several studies have indicated that lymph node status may have less prognostic impact in salvage esophagectomy than in primary surgery [43,45,46]. Furthermore, limited lymphadenectomy does not appear to adversely affect long-term oncological outcomes [52,53]. Consequently, a more selective strategy targeting only lymph nodes suspected of metastasis based on pre-treatment or preoperative evaluation is increasingly being adopted. Nevertheless, robust evidence remains limited, and further validation is warranted.

Preservation of the bronchial arteries is critical to reduce the risk of tracheobronchial ischemia and necrosis [38]. In addition to meticulous dissection around the airway, extensive cervical lymph node dissection should be avoided whenever possible in order to preserve blood flow from the inferior thyroid artery to the trachea. Accordingly, excessive dissection around the trachea and bronchi, including subcarinal lymph node dissection, should also be avoided [38,54]. Once tracheobronchial ischemia or necrosis occurs, prompt control of aspiration pneumonia becomes the primary therapeutic priority. In cases associated with anastomotic leakage, removal of the reconstructive conduit with creation of an esophagostomy is often required. Simultaneously, closure of the airway defect using a pedicled muscle flap, such as the latissimus dorsi, pectoralis major, or intercostal muscle, should be performed [38,54].

Reconstruction strategies also require careful consideration. The posterior mediastinal route may not be optimal in salvage settings because prior irradiation may increase the risk of anastomotic leakage [37,38,44,45,46,47]. Therefore, alternative routes such as the retrosternal pathway are often preferred. Intraoperative assessment of gastric conduit perfusion has also been increasingly adopted to reduce anastomotic complications [55,56]. In cases of inadequate perfusion, staged reconstruction should be considered.

### 7.5. Oncological Outcomes

The oncological outcomes of salvage esophagectomy depend on multiple factors, including tumor stage, response to dCRT, and completeness of resection.

Patients with residual disease after dCRT generally have worse outcomes than those with recurrence after achieving a complete response, likely reflecting intrinsic resistance to chemoradiotherapy [43,45,46]. Reported 5-year overall survival rates after salvage esophagectomy range from 20% to 40% overall but may exceed 40–60% in patients with early-stage (T1–2) disease [37,45,46]. However, salvage esophagectomy for advanced residual disease may not always provide sufficient oncological radicality. The greatest survival benefit appears to be achieved in patients with residual tumor confined to the adventitial layer and in those with resectable locoregional recurrence.

## 8. Local Salvage Therapies

In addition to salvage esophagectomy, several less invasive local salvage therapies have been developed for the management of residual or recurrent lesions after dCRT. Among these, endoscopic submucosal dissection (ESD) and photodynamic therapy (PDT) are the most commonly employed modalities. These approaches are particularly attractive for patients with limited disease and high surgical risk because they offer the potential for local tumor control while avoiding the substantial morbidity associated with salvage esophagectomy.

### 8.1. ESD

ESD is primarily indicated for superficial residual or recurrent lesions confined to the mucosal or superficial submucosal layers. Owing to its ability to achieve en bloc resection, ESD enables precise histopathological evaluation and has demonstrated favorable local control and resection outcomes [57]. Although ESD offers the advantage of organ preservation with relatively low procedure-related mortality, its curative potential remains limited, with reported 3-year recurrence-free survival rates ranging from 49% to 58% [58,59].

However, post-dCRT tissues frequently exhibit marked fibrosis and scarring, which obscure normal submucosal planes and make dissection technically challenging. Consequently, ESD in this setting is generally more difficult than in treatment-naïve cases and may be associated with an increased risk of complications such as perforation and bleeding [57,58,59]. These limitations highlight the need for alternative local salvage modalities, particularly for lesions with deeper invasion or technically difficult anatomy.

### 8.2. PDT

**Figure 4:**
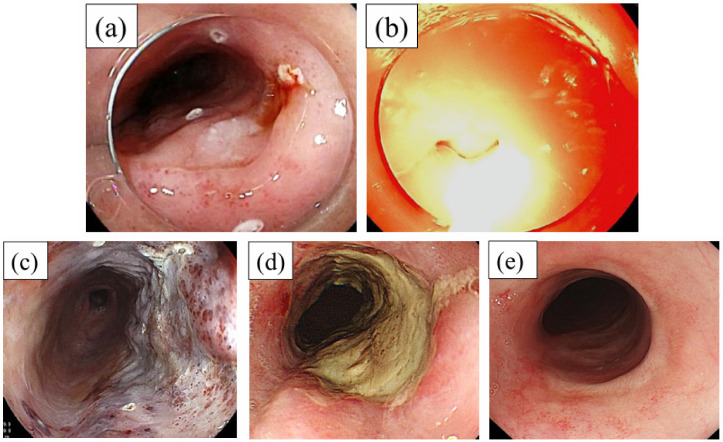
Photodynamic Therapy (PDT). (**a**) esophageal tumor before PDT, cT1b lesion at the lower esophagus, (**b**) laser irradiation using diode laser at 664 nm wavelength. (**c**) 1 day after PDT, the treated mucosal area shows only grayish-white discoloration within the irradiated field. (**d**) 7 days after PDT, the treated area has developed an ulcerative lesion, and the ulcer is covered with necrotic tissue. (**e**) 2 months after PDT, the treated area appears firm and fibrotic, but is completely covered by regenerated epithelium.

PDT has emerged as an important local salvage modality for ESCC, particularly in patients with residual or recurrent disease after dCRT. PDT involves administration of talaporfin sodium, a photosensitizing agent, followed by targeted laser irradiation, which induces selective tumor necrosis through photochemical reactions [60] (Figure 4). PDT is generally indicated for residual or recurrent thoracic esophageal cancer after dCRT, particularly in patients with superficial to moderately deep lesions (typically T1–T2) without distant metastasis. This approach is especially useful when endoscopic resection is technically difficult because of fibrosis or when patients are not suitable candidates for salvage surgery. In addition, the indication for PDT is usually limited to lesions measuring ≤ 3 cm in longitudinal length and involving no more than half of the esophageal circumference [60,61].

The principal advantage of PDT lies in its minimally invasive nature and its ability to preserve the esophagus while achieving local tumor control. Reported complete response rates range from approximately 60% to 80% in appropriately selected patients [60,61,62]. However, PDT also has several limitations, including the risk of esophageal stricture, skin phototoxicity, and incomplete tumor eradication, particularly in lesions with deeper invasion [60,61,62]. Nevertheless, PDT remains a valuable salvage option in carefully selected patients.

## 9. Immunotherapeutic Salvage Treatment After dCRT

For patients with residual or recurrent ESCC after dCRT who are not candidates for local salvage therapies such as ESD, PDT, or salvage esophagectomy, systemic immunotherapy has recently emerged as a promising treatment option. ICIs, particularly those targeting the programmed death-1 (PD-1) pathway, have demonstrated significant clinical efficacy in advanced or unresectable esophageal cancer [7,8] and have also shown potential therapeutic value in the salvage setting [34,63].

Accumulating evidence suggests a potential synergistic interaction between radiotherapy and immunotherapy. Radiation may enhance tumor antigen presentation and modulate the tumor microenvironment, thereby augmenting systemic antitumor immune responses—a phenomenon referred to as the abscopal effect [64]. In the context of dCRT, prior radiation exposure may therefore enhance the efficacy of subsequent immunotherapy in salvage treatment. Although prospective data specifically evaluating ICIs as salvage therapy after dCRT remain limited, ongoing clinical trials are expected to further clarify their role [34]. Taken together, immunotherapeutic approaches are becoming an increasingly important component of salvage treatment strategies for ESCC, particularly in patients who are not suitable candidates for local or surgical interventions.

## 10. Palliative Esophageal Stenting and Radiation Therapy

**Figure 5:**
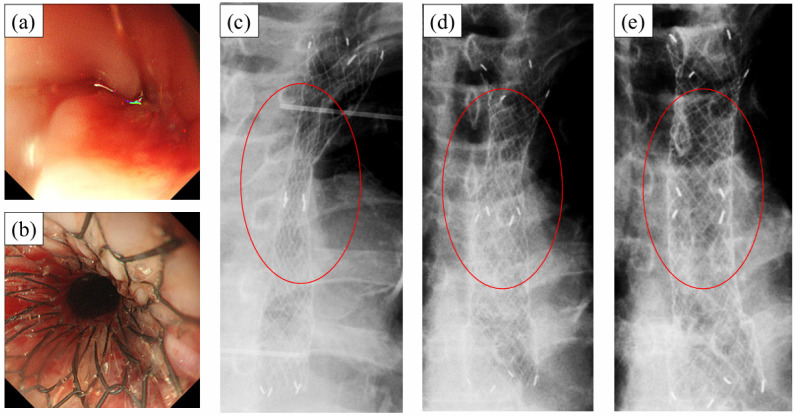
Esophageal stenting to the case after definitive chemoradiotherapy (dCRT). (**a**) esophageal stenosis due to recurrence of tumor after dCRT (**b**) Endoscopic findings immediately after self-expanding metal stents (SEMS) placement. Although the esophageal lumen became visible, the stent showed only minimal expansion. (**c**) Chest radiograph obtained on the same day after SEMS placement (red circle) showing persistent severe luminal narrowing due to incomplete stent expansion. (**d**) 1 day after stenting, substantial stent expansion, although residual waist formation remained at the stenotic segment. (**e**) 7 days after stenting, complete resolution of the stenosis with full stent expansion.

Palliative esophageal stenting plays a crucial role in the management of dysphagia in patients with advanced or recurrent esophageal cancer, including those previously treated with radiotherapy or dCRT (Figure 5). Stenting provides immediate symptom relief and improves oral intake, thereby enhancing quality of life. However, the use of esophageal stents after radiotherapy was historically considered hazardous because of the high risk of severe complications. Radiation-induced tissue damage, including fibrosis, reduced vascularity, and impaired healing capacity, increases susceptibility to adverse events, particularly esophageal perforation and hemorrhage [65,66]. This concern is also reflected in current guidelines, which recognize prior radiotherapy as a risk factor for stent-related adverse events, although stent placement is not contraindicated in this setting [67]. Nevertheless, with the development of modern self-expanding metal stents (SEMS), procedural outcomes and safety profiles have improved substantially, and stent placement may be considered in selected patients with prior irradiation [68], although careful risk assessment remains essential.

The combination of stenting and palliative radiotherapy remains an area of ongoing debate. Although radiotherapy may provide more durable tumor control and dysphagia relief, radiotherapy administered before stenting may compromise tissue integrity and increase procedural risk. A randomized controlled trial reported that patients with advanced esophageal cancer who underwent SEMS placement for primary palliation of dysphagia did not derive additional benefit from concurrent palliative radiotherapy [69]. Consistent with these findings, the ESGE Guideline (2021) does not recommend the routine addition of radiotherapy following SEMS placement [67]. Conversely, more recent retrospective studies have suggested that stent placement followed by radiotherapy is not associated with an increased risk of high-grade complications compared with stenting alone and may be associated with prolonged survival [68,70]. However, these findings are based on retrospective analyses and should therefore be interpreted with caution. Thus, the optimal sequencing of these modalities has yet to be clearly defined.

## 11. Conclusions

**Figure 6:**
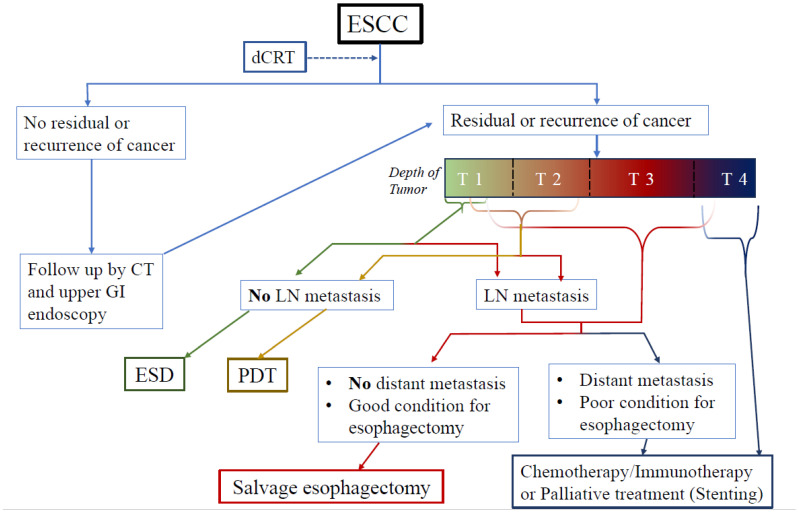
Proposed treatment algorithm for residual or recurrent esophageal squamous cell carcinoma after definitive chemoradiotherapy (dCRT).

The management of residual or recurrent ESCC after dCRT requires a structured and individualized treatment strategy based on tumor depth, nodal and distant metastatic status, and patient operability (Figure 6). As summarized in the proposed treatment algorithm, local salvage therapies such as ESD and PDT may be appropriate for superficial disease, whereas salvage esophagectomy remains the principal curative option for resectable locally advanced tumors. In patients with unresectable disease or poor surgical tolerance, systemic therapy including immunotherapy, as well as palliative interventions, should be considered.

Given the complexity of post-radiation disease and treatment-related morbidity, a multidisciplinary and individualized approach is essential to optimize clinical outcomes. Further prospective studies are warranted to refine treatment algorithms and improve long-term survival in this challenging clinical setting.

Treatment selection is based on tumor depth, nodal and distant metastatic status, and surgical tolerance. Endoscopic submucosal dissection (ESD) or photodynamic therapy (PDT) may be considered for superficial lesions, whereas salvage esophagectomy remains the primary curative option for resectable locally advanced disease. In unresectable cases or patients unfit for surgery, systemic therapy including chemotherapy and immunotherapy, as well as palliative interventions such as esophageal stenting, should be considered. The detailed indications, advantages, limitations, and patient selection criteria for each salvage modality are discussed in the relevant sections of this review.

## Figures and Tables

**Table 1:** Summary of landmark clinical trials investigating definitive chemoradiotherapy and multimodal treatment strategies in esophageal squamous cell carcinoma (ESCC). CRT, chemoradiotherapy; dCRT, definitive chemoradiotherapy; CF, cisplatin plus 5-fluorouracil; DCF, docetaxel, cisplatin, and 5-fluorouracil; RT, radiotherapy; OS, overall survival; PFS, progression-free survival; EFS, event-free survival; CR, complete response; vs., versus.

Trial	Population	Treatment Protocol	Key Outcomes	Conclusion
JCOG9708	Stage I ESCC	5-FU + cisplatin × 2 + RT 60 Gy (split-course)	CR 87.5%; 4-year OS 80.5%	CRT showed excellent efficacy and became a standard option for Stage I ESCC
JCOG0502	Stage I ESCC	Surgery vs. CRT (CF + RT)	5-year OS 85.5% comparable between surgery and CRT	CRT was non-inferior and established as an organ-preserving alternative
JCOG0909	Stage II/III thoracic ESCC	CF-based dCRT + elective nodal irradiation + salvage treatment	CR 59% 3-year OS 74%	Validated dCRT with planned salvage as a treatment strategy
JCOG1510	Locally advanced unresectable thoracic ESCC	Induction DCF → conversion surgery or dCRT vs. standard dCRT	Ongoing primary endpoint OS	-
KEYNOTE-975	Locally advanced unresectable esophageal cancer (ESCC/EAC)	dCRT (FP or FOLFOX + RT 50–60 Gy) ± pembrolizumab	Ongoing primary endpoints: OS, EFS	-
EC-CRT-002	Unresectable locally advanced ESCC	Induction chemotherapy + tislelizumab → concurrent CRT ± maintenance tislelizumab	Improved PFS and OS with manageable toxicity	Supports immunotherapy-based intensification in dCRT

## Data Availability

No new data were created or analyzed in this study. Data sharing is not applicable to this article.
